# Eight-Shaped Hatching Increases the Risk of Inner Cell Mass Splitting in Extended Mouse Embryo Culture

**DOI:** 10.1371/journal.pone.0145172

**Published:** 2015-12-17

**Authors:** Zheng Yan, Hongxing Liang, Li Deng, Hui Long, Hong Chen, Weiran Chai, Lun Suo, Chen Xu, Yanping Kuang, Lingqian Wu, Shengsheng Lu, Qifeng Lyu

**Affiliations:** 1 Department of Assisted Reproduction, Shanghai Ninth People’s Hospital Affiliated to Shanghai Jiao Tong University School of Medicine, Shanghai 200011, PR China; 2 Guangxi High Education key Laboratory for Animal Reproduction and Biotechnology, State Key Laboratory for Conservation and Utilization of Subtropical Agro-Bioresources, Guangxi University, Nanning, 530004, PR China; 3 National Laboratory of Medical Genetics, Xiangya Hospital, Central South University, Changsha, 410078, PR China; 4 Shanghai Key Laboratory of Reproductive Medicine, Shanghai 200025, PR China; Michigan State University, UNITED STATES

## Abstract

Increased risk of monozygotic twinning (MZT) has been shown to be associated with assisted reproduction techniques, particularly blastocyst culture. Interestingly, inner cell mass (ICM) splitting in human ‘8’-shaped hatching blastocysts that resulted in MZT was reported. However, the underlying cause of MZT is not known. In this study, we investigated in a mouse model whether in vitro culture leads to ICM splitting and its association with hatching types. Blastocyst hatching was observed in: (i) in vivo developed blastocysts and (ii–iii) in vitro cultured blastocysts following in vivo or in vitro fertilization. We found that ‘8’-shaped hatching occurred with significantly higher frequency in the two groups of in vitro cultured blastocysts than in the group of in vivo developed blastocysts (24.4% and 20.4% versus 0.8%, respectively; n = 805, *P* < 0.01). Moreover, Oct4 immunofluorescence staining was performed to identify the ICM in the hatching and hatched blastocysts. Scattered and split distribution of ICM cells was observed around the small zona opening of ‘8’-shaped hatching blastocysts. This occurred at a high frequency in the in vitro cultured groups. Furthermore, we found more double OCT4-positive masses, suggestive of increased ICM splitting in ‘8’-shaped hatching and hatched blastocysts than in ‘U’-shaped hatching and hatched blastocysts (12.5% versus 1.9%, respectively; n = 838, *P* < 0.01). Therefore, our results demonstrate that extended in vitro culture can cause high frequencies of ‘8’-shaped hatching, and ‘8’-shaped hatching that may disturb ICM herniation leading to increased risk of ICM splitting in mouse blastocysts. These results may provide insights into the increased risk of human MZT after in vitro fertilization and blastocyst transfer.

## Introduction

Monozygotic twinning (MZT) is an uncommon phenomenon that occurs in about 0.4–0.45% of natural in vivo conceptions [1]. However, the etiology of MZT is not clear. In recent years, the use of assisted reproductive technology (ART) has been reported to increase the incidence of MZT by 2–12-fold compared with that of natural conception [2–4]. Moreover, MZT is associated with a high risk of perinatal mortality and congenital anomalies [5, 6]. The factors that contribute to the increased frequency of MZT following ART are not entirely clear. Because of our lack of understanding of the mechanisms of embryo splitting, several factors, including ovulation induction [7, 8], micromanipulation of the oocyte or embryo [9, 10], and multiple gestations [11, 12], have been proposed to affect the occurrence of MZT. Interestingly, when embryos are cultured to the blastocyst stage before embryo transfer, the incidence of MZT pregnancy increases dramatically [13, 14]. Therefore, extended embryo culture is one of the primary factors of ART most frequently associated with MZT.

Blastocyst culture is now performed in many ART centers worldwide to select for well-developed embryos or normal embryos by pre-implantation genetic screening before embryo transfer. However, blastocyst culture has been shown to be associated with a significantly elevated risk of MZT, suggesting that prolonged in vitro culture may induce splitting of the embryo or the inner cell mass (ICM) [15, 16]. Many researchers have proposed explanations for the ICM splitting observed during blastocyst development, including the blastomere herniation hypothesis, delayed implantation, cell apoptosis theory, and interruption of cell-to-cell adhesion [17–20]. However, none of these provide a complete explanation. In addition, two case reports have shown that human blastocysts with ‘8’-shaped hatching, which herniated from small openings in the zona pellucida, resulted in double ICM-like masses in two blastocoels [21, 22], and one MZT pregnancy [21]. However, in these cases, it is still unknown whether these double masses were actually split ICMs and whether ICM splitting in these cases was associated with the ‘8’-shaped hatching. It is a very interesting question that: Are these cases just happenchance or events which can be recapitulated under some certain conditions? It is presumed that ‘8’-shaped hatching from artificial zona opening, such as assisted hatching and intracytoplasmic sperm injection, should induce mechanical ICM splitting [23, 24]. Does the extended in vitro culture increase ‘8’-shaped hatching and then lead to ICM splitting?

Because of the infrequent occurrence of MZT, most studies have provided theoretical assumptions based on clinical data analysis or case reports to describe this phenomenon. Large-scale animal experiments are lacking, however, such studies are required to confirm the occurrence of ICM splitting and illustrate the mechanism of increased MZT occurrence during extended in vitro culture. In this study, we attempt to recreate the phenomena of human ‘8’-shaped hatching with ICM-like splitting [21, 22] by in vitro experimental verification in a mouse model. We test whether extended in vitro culture would cause more ‘8’-shaped hatching, as well as whether the ‘8’-shaped hatching would lead to ICM splitting. Our results demonstrate that in vitro culture of in vivo and in vitro fertilized embryos causes a significant increase in ‘8’-shaped hatching and ICM splitting. These results provide new insights into the origin of ICM splitting during blastocyst culture and may provide a potential explanation for the increased occurrence of human MZT when transferring in vitro-produced blastocysts.

## Materials and Methods

### Collection of mouse oocytes, zygotes and embryos

Kun Ming Bai (KMB) mice from the SLAC Laboratory Animal Co. Ltd (Shanghai, PRC) were used in our study to collect oocytes, spermatozoa and embryos. All mice were maintained under specific pathogen-free (SPF) conditions and were housed in sterilized cages containing sterilized feed, autoclaved bedding, and water at 20 temperature and 40% humidity condition in the Experimental Animal Centre of Shanghai Ninth People’s Hospital. All the experimental procedures were approved by the Animal Ethics Committee at the Shanghai Ninth People’s Hospital and carried out in strict accordance with the recommendations in the Guide for the Care and Use of Laboratory Animals of Shanghai Jiao Tong University School of Medicine, China. All mice were sacrificed under isoflurane anesthesia, and all efforts were made to minimize suffering. All female KMB mice (4–6 weeks of age) were injected with 10 IU pregnant mare serum gonadotropin (PMSG; Tianjin Animal Hormone Factory, Tianjin City, China), followed by 10 IU human chorionic gonadotropin (HCG; Ningbo Animal Hormone Factory, Ningbo City, China) 46–48 h later.

Mouse blastocysts were divided into three groups: (i) one in vivo developed group, and two in vitro cultured groups, (ii) in vivo fertilization and embryo culture group and (iii) in vitro fertilization and embryo culture group. For the in vivo developed group, super-ovulated female mice were mated with males. The day on which vaginal plugs were observed was calculated as day 0.5. Embryos were collected by tubal flushing on day 4, and expanded blastocysts were chosen for hatching observation. For the in vivo fertilization and in vitro culture group, female mice mated with males were sacrificed 18–20 h after HCG injection. Zygotes with two pronuclei and extrusion of the second polar body observed under a microscope were cultured in vitro. The expanded blastocysts were selected on day 4. Finally, for the in vitro fertilization and embryo culture group, oocytes were collected from the oviducts 13–15 h after HCG injection. Sperm from the caudal epididymis of adult male KMB mice (10–14 weeks of age) were suspended in human tubal fluid (HTF) medium (Millipore, Billerica, MA, USA) and incubated at 37°C under 5% CO_2_ and 95% humidity for 1.5 h. The capacitated sperm suspension was gently added to the cumulus-oocyte complexes at a final motile sperm concentration of 1–2 × 10^6^ cells/mL. The oocytes and sperm were incubated in HTF medium for fertilization. After 4–6 h, the fertilized oocytes were washed three times and transferred to fresh culture medium. Double pronuclei were confirmed with phase-contrast microscopy. Expanded blastocysts were observed during the hatching process.

### Embryo culture and observation

Mouse zygotes and embryos were cultured in KOSM medium (Millipore) at 37°C in an atmosphere containing 5% CO_2_. Expanded blastocysts that formed at day 4 post-fertilization with normal morphology of ICM and trophectoderm were used for observation of the hatching process until day 5 post-fertilization or later. Culture beyond four or five days were called as extended in vitro culture, which are groups (ii) and (iii). All embryos were cultured in microdrops of medium in tissue culture dishes (Falcon 3001; Becton Dickinson, USA) overlaid with mineral oil (Irvine Scientific, USA). Blastocysts were observed and photographed two or three times a day. The sizes of zona openings were carefully observed and evaluated using Image-Pro Plus 6.0 software. Blastocysts that maintained two blastocoels with nearly equal size and with hatching opening sizes of less than 25 μm beyond 12 h of observation were identified as ‘8’-shaped hatching blastocysts with small hatching openings. Blastocysts hatching with one blastocoel and a hatching opening size that increased to more than 25 μm within 12 h of observation were identified as ‘U’-shaped hatching blastocysts with large hatching openings.

### Immunofluorescence staining and confocal laser-scanning microscopy

Embryos at day 5 post-fertilization were fixed directly in 4% paraformaldehyde for 30 min at room temperature. Embryos were then transferred through several washes of 1× phosphate-buffered saline (PBS) plus 0.1% Triton-100 (PBS/0.1%T) to remove residual paraformaldehyde. Embryos were placed in PBS/0.1%T for 30 min at room temperature for permeabilization. After washing three times with blocking solution (PBS containing 3% goat serum), embryos were blocked for 1 h at room temperature. Embryos were then incubated with anti-OCT4 antibodies (Sigma-Aldrich, St. Louis, MO, USA) diluted 1:100 in blocking solution overnight at 4°C. The following day, after three 15-min washes in blocking solution, embryos were incubated with secondary antibodies (Alexa Fluor 488 dye; Jackson ImmunoResearch Laboratories, USA) diluted 1:200 in blocking solution for 30 min at room temperature. Following another three washes, chromatin was stained with Hoechst33342 (Sigma-Aldrich) for 5 min at room temperature. Finally, the stained embryos were suspended in a microdrop with blocking solution and photographed with a confocal laser-scanning microscope (Zeiss LSM 710 Meta; Carl Zeiss AG, Oberkochen, Germany). OCT4-positive cell masses were identified as ICMs.

### Statistical analysis

Data were analyzed by Pearson’s chi-square tests, continuity correction chi-square tests, and Fisher's exact tests using SPSS 16.0 software. Differences with *P* values of less than 0.05 were considered statistically significant.

## Results

### Extended in vitro culture causes high frequency of ‘8’-shaped hatching

**Fig 1:**
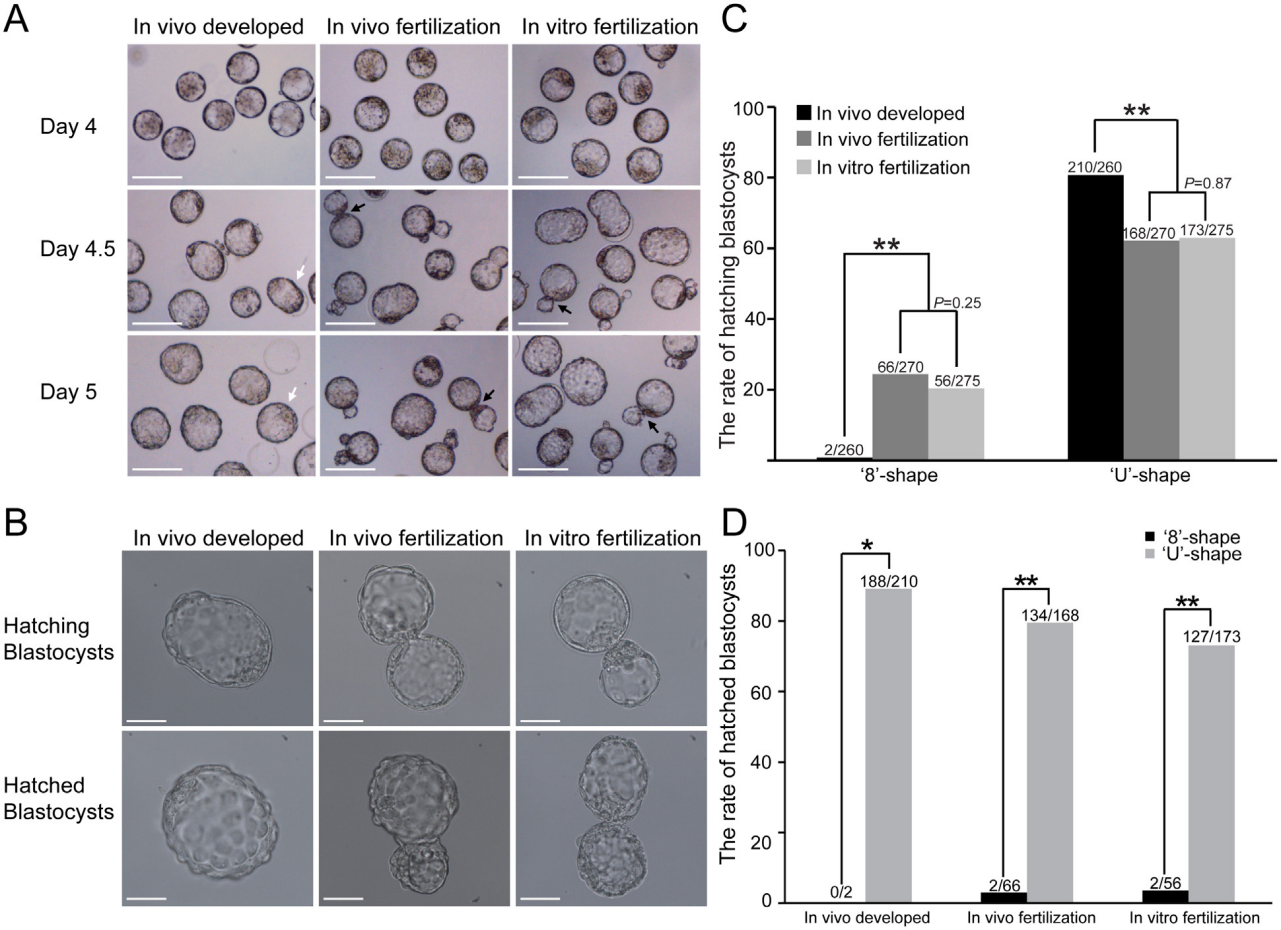
The frequency of ‘8’-shaped hatchings in mouse blastocysts was higher in in vitro cultured groups than the in vivo developed group. (A) Representative images of pre-implantation embryos show the dynamic hatching process at day 4 (expanded blastocysts), day 4.5 (hatching blastocysts), and day 5 (hatched blastocysts) after fertilization. White arrows indicate ‘U’-shaped hatching blastocysts with large openings. Black arrows indicate ‘8’-shaped hatching blastocysts. The scale bar is 100 μm. (B) Images of ‘U’-shaped hatching and hatched blastocysts with large zona openings in the in vivo developed group (left panel) and ‘8’-shaped hatching and hatched blastocysts in both in vitro cultured groups (i.e., the in vivo fertilization group and the in vitro fertilization group, middle and right panels, respectively). The scale bar is 50 μm. (C) Comparison of hatching blastocysts exhibiting ‘8’-shaped and ‘U’-shaped hatching in the three groups. All the percentages are fractions based on the total number of expanded blastocysts for each group. **P < 0.01. (D) Comparison of hatched blastocysts exhibiting ‘8’-shaped and ‘U’-shaped hatching in the three groups. All the percentages are fractions based on the total number of hatching blastocysts for each group. *P < 0.05, **P < 0.01.

To evaluate the effects of in vitro culture on the blastocyst hatching process, 805 expanded blastocysts with normal morphology from three different groups were selected for observation. The results showed high frequencies of ‘8’-shaped hatching blastocysts in both in vitro cultured groups. However, most of the expanded blastocysts in the in vivo developed group underwent ‘U’-shaped hatching during 1 day of in vitro culture (Fig 1A). Only two ‘8’-shaped hatching blastocysts were found in the in vivo developed group. The ‘8’-shaped hatching rate in this group was significantly lower than those of both in vitro cultured groups (0.8% versus 24.4% and 20.4%, respectively; *P* < 0.01; Fig 1C and Table 1). In contrast, the ‘U’-shape hatching rate in the in vivo developed group was higher than those in the other two groups (80.8% versus 62.2% and 62.9%, respectively; *P* < 0.01; Fig 1C and Table 1). Furthermore, we found that the blastocysts that underwent ‘8’-shaped hatching struggled to achieve complete hatching due to the small zona opening. Only 3% (2/66) and 3.6% (2/56) of the ‘8’-shaped blastocysts hatched out completely in both in vitro cultured groups. The hatched rate of the ‘8’-shaped blastocysts in each group was significantly lower than that of the ‘U’-shaped blastocysts in the same group (*P* < 0.05; Fig 1D and Table 2). Interestingly, the few hatched blastocysts from the small hatching opening maintained the ‘8’-shape with two half-blastocoels (Fig 1B). These results demonstrated that prolonged in vitro culture could cause a high frequency of ‘8’-shaped hatching blastocysts owing to the small hatching opening in the zona pellucida, which had adverse effects on the hatching process.

**Table 1:** Comparison of hatching types in mouse blastocysts from three groups with different embryo derivation.

Group	No. of expanded blastocysts	No. of ‘8’-shape hatching blastocysts (%)	No. of ‘U’-shape hatching blastocysts (%)	No. of non-hatching blastocysts (%)
In vivo developed	260	2 (0.8) ^a^ ^,^ ^b^	210 (80.8) ^c^ ^,^ ^d^	48 (18.4)
in vivo fertilization and embryo culture	270	66 (24.4) ^a^	168 (62.2) ^c^	36 (13.4)
in vitro fertilization and embryo culture	275	56 (20.4) ^b^	173 (62.9) ^d^	46 (16.7)

All the percentages are the fractions based on the total number of expanded blastocysts from each group.

^a, b, c, d^ Values with the same superscripts are significantly different (*P* < 0.01, χ^2^-test).

**Table 2:** Comparison of hatched rates between ‘8’-shape and ‘U’-shape hatching blastocysts.

	In vivo developed group %		In vivo fertilization and embryo culture %		In vitro fertilization and embryo culture %	
Hatching type	Hatched	Un-hatched	Hatched	Un-hatched	Hatched	Un-hatched
‘8’-shape	0 (0/2) ^a^	100 (2/2)	3 (2/66) ^b^	97 (64/66)	3.6 (2/56) ^c^	96.4 (54/56)
‘U’-shape	89.5 (188/210) ^a^	10.5 (22/210)	79.8 (134/168) ^b^	20.2 (34/168)	73.4 (127/173) ^c^	26.6 (46/173)

‘8’-shape = ‘8’-shaped hatching blastocyst with small opening of zona pellucida; ‘U’-shape = ‘U’-shaped hatching blastocyst with large opening of zona pellucida. Hatched = the blastocyst finished hatching and escaped the zona pellucida. Un-Hatched = the blastocyst started hatching but did not escape the zona pellucida. All the percentages are the fractions based on the total number of ‘8’-shaped or ‘U’-shaped hatching blastocysts of each group.

^a, b, c^ Values with the same superscripts are significantly different (*P* < 0.05, χ^2^-test).

### ‘8’-shaped hatching led to the increased occurrence of double ICMs with positive staining for OCT4 expression in mouse blastocysts

**Fig 2:**
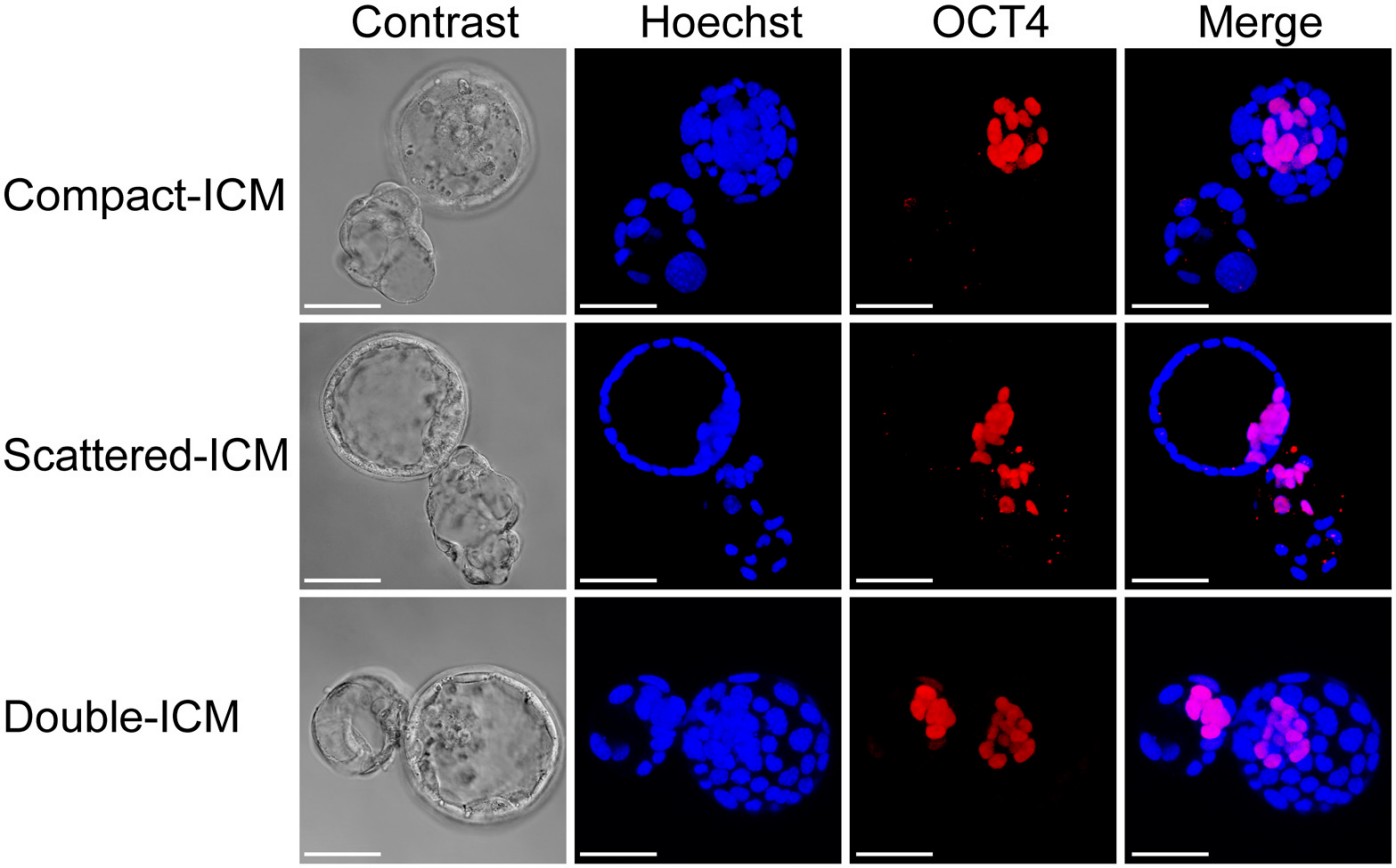
Immunofluorescence staining of OCT-4 in ‘8’-shaped hatching blastocysts. Representative images showing the different shapes and distributions of ICMs in mouse ‘8’-shaped hatching blastocysts: compact ICMs were far away from zona opening (upper panel); scattered ICMs were around the small zona opening (middle panel); double ICMs with two obviously separated masses divided into each of the herniated blastocoels and were around the small zona opening (lower panel). ICMs were stained with anti-OCT-4 antibodies (red). All nuclei were labeled with Hoechst 33342 (blue). The scale bar is 50 μm.

**Fig 3:**
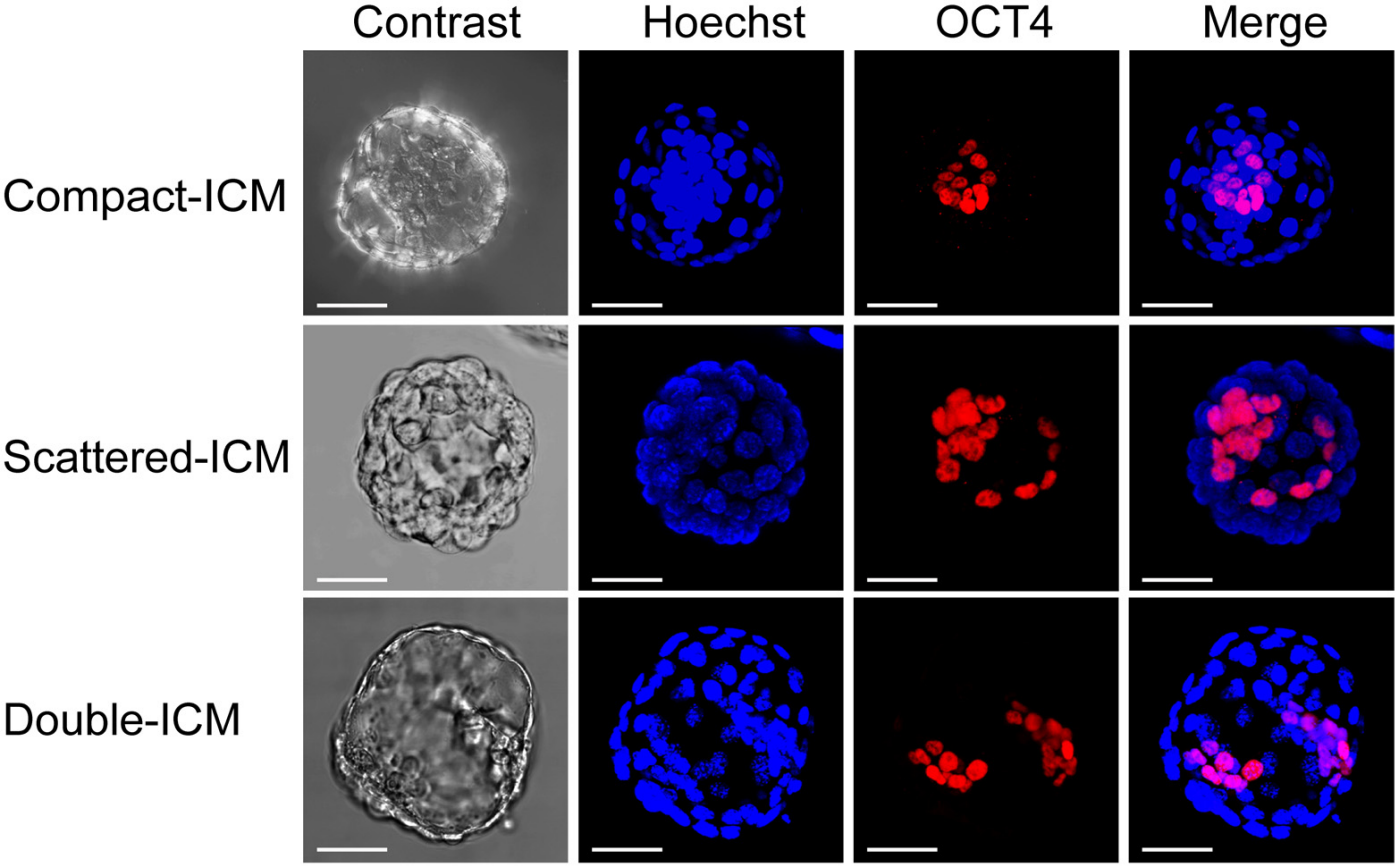
Immunofluorescence staining of OCT-4 in completely hatched blastocysts through ‘U’-shaped hatching. Representative images showing the different shapes and distributions of ICMs in mouse ‘U’-shaped hatching and hatched blastocysts: compact ICMs (upper panel), scattered ICMs (middle panel), and double ICMs in one blastocoel (lower panel). ICMs were stained with anti-OCT-4 antibodies (red). All nuclei were labeled with Hoechst 33342 (blue). The scale bar is 50 μm.

Firstly, we investigated the effect of embryo culture on the distribution of ICMs in the hatching and hatched blastocysts from three groups by detecting the OCT4 expression with immunofluorescence staining. Double ICMs were found in both in vitro culture groups, whereas none were detected in in vivo developed group (3.9% and 3.5% versus 0%, *P* < 0.01, Table 3). Next, we studied the effects of different hatching types on the distribution of ICMs. 312 ‘8’-shaped and 526 ‘U’-shaped blastocysts from in vitro culture groups were analyzed using immunofluorescence staining for OCT4. Double ICMs with positive staining for OCT4 expression were found in 12.5% (39/312) of ‘8’-shaped hatching blastocysts (Fig 2, lower panel). Only 10 double ICMs in one blastocoel were detected in 526 hatched blastocysts that underwent ‘U’-shaped hatching (Fig 3, lower panel). The rate of double ICMs in the ‘8’-shaped hatching blastocysts was significantly higher than that in the ‘U’-shaped hatching blastocysts (12.5% versus 1.9%, respectively; *P* < 0.01; Table 4). Additionally, 23.1% (72/312) of ICMs from ‘8’-shaped blastocysts presented abnormal shapes, such as scattered or dispersed ICMs, rather than the typical double ICMs (Fig 2, middle panel). This rate was also significantly higher than that in the ‘U’-shaped hatching blastocysts (*P* < 0.01; Fig 3, middle panel and Table 4). Such scattered or split ICM located around the small zona opening of ‘8’-shaped hatching blastocyst. In addition, 64.4% (201/312) and 83.3% (438/526) of ICMs in the ‘8’-shaped and ‘U’-shaped hatching blastocysts, respectively, were of normal shape, exhibiting one compact cell mass with positive staining for OCT4 expression (*P* < 0.01; Figs 2 and 3, upper panel and Table 4). Such compact ICM in ‘8’-shaped hatching blastocysts located far away from the small zona opening. Therefore, the ‘8’-shaped hatching in blastocysts increased the occurrence of abnormal distribution of ICM and double ICMs. These results suggested that the ICM was more sensitive to disruption or splitting in ‘8’-shaped hatching blastocysts.

**Table 3:** Distribution of ICMs in three groups with different embryo derivation.

Group	In vivo developed (%)	In vivo fertilization and embryo culture (%)	In vitro fertilization and embryo culture (%)
No. of blastocysts	247	203	257
No. of HB with compact ICM	214 (86.6) ^a^ ^,^ ^b^	153 (75.4) ^a^	195 (75.9) ^b^
No. of HB with scattered ICM	33 (13.4) ^c^ ^,^ ^d^	42 (20.7) ^c^	53 (20.6) ^d^
No. of HB with double ICM	0 (0) ^e^ ^,^ ^f^	8 (3.9) ^e^	9 (3.5) ^f^

*Note*: HB = hatching and hatched blastocysts, ICM = inner cell mass. All the percentages are based on the staining number of hatching and hatched blastocysts of each group.

^a, b, c, d, e, f^ Values with same superscripts are significantly different (*P* < 0.05, χ^2^-test)

**Table 4:** Distribution of ICMs between ‘8’-shaped and ‘U’-shaped blastocysts.

	HB with ‘8’-shape (%)	HB with ‘U’-shape (%)
No. of blastocysts	312	526
No. of HB with compact ICM	201 (64.4)^a^	438 (83.3)^a^
No. of HB with scattered ICM	72 (23.1)^b^	78 (14.8)^b^
No. of HB with double ICM	39 (12.5)^c^	10 (1.9)^c^

*Note*: HB = hatching and hatched blastocysts, ICM = inner cell mass. All the percentages are based on the staining number of hatching and hatched blastocysts of each group.

^a, b, c^Values with same superscripts are significantly different (*P* < 0.01, χ^2^-test)

## Discussion

In the present study, we investigated for the first time, the relationship among the in vitro culture, hatching types of blastocysts and ICM splitting. Observations in a large number of mouse in vivo derived blastocysts and in vitro cultured blastocysts showed that more than 20% of in vitro cultured hatching blastocysts underwent ‘8’-shaped hatching. This rate was significantly higher than that in the in vivo developed blastocysts proceeding only 1 day of in vitro culture. These data demonstrate that extended embryo culture can significantly increase the occurrence of ‘8’-shaped hatching. Furthermore, we found that there was a higher frequency of double ICMs confirmed by OCT4-positive staining in ‘8’-shaped hatching blastocysts than in ‘U’-shaped hatching blastocysts. Scattered or split distribution of OCT4-positive masses was found around the small zona opening of ‘8’-shaped hatching. Whereas, compact distribution of OCT4-positive masses was found in ‘U’-shaped hatching blastocysts or far away from the small zona opening in ‘8’-shaped hatching blastocysts. These phenomena indicate the affection of small zona opening on the cells distribution of ICM. ICM herniation would be ruptured or split by the trapping of small zona opening and the dragging of hatching force. These findings suggested that ICMs were more susceptible to disturbances when blastocysts were hatched from small openings of the zona pellucida. Nevertheless, a few blastocysts exhibiting ICM splitting also occurred in the ‘U’-shaped hatching group, indicating that the ‘8’-shaped hatching was an important factor, but not the only factor causing ICM splitting.

Extended embryo culture has been shown to cause the zona pellucida to harden [25]. In our study, however, we focused on the opening size of the zona pellucida rather than the hardness of the zona pellucida because we assumed that hardening of the zona during in vitro culture would finally influence the hatching opening of the zona pellucida. This is the first experimental research describing the association between the zona opening from extended in vitro culture and embryo splitting. The alteration in the zona architecture from prolonged in vitro culture should make it more difficult for the expanded blastocyst to break through the zona pellucida, thereby increasing the occurrence of small hatching openings and then leading to ‘8’-shaped hatchings. Our data showed that the ‘8’-shaped hatching rates from both in vitro cultured groups were similar, but were significantly higher than that from the in vivo developed group. However, the similar results of ‘8’-shaped hatching rates in both in vitro cultured groups indicated that in vitro fertilization did not affect the hatching opening size of the zona pellucida. Furthermore, we found that the frequency of double-ICMs was significantly higher in the ‘8’-shaped hatching blastocysts than in the ‘U’-shaped hatching blastocysts. These results demonstrated that changes in the zona pellucida resulting from prolonged exposure to culture media before blastocyst transfer could cause pinching of the ICM during the hatching process, which may cause ICM splitting.

MZT occurs rarely in mice [26], however, ICMs have been shown to split into two separate groups of cells in mouse embryos cultured in vitro at the early blastocyst stage and then to develop and subdivide into two independent egg cylinders after hatching from the zona pellucida and attaching to the feeder layer [27]. Double ICMs have also been shown to occur in the early blastocyst stage prior to hatching during extended in vitro culture at rates of 0.6% and 3.1% for in vivo and in vitro fertilization, respectively [28]. These findings illustrated that the formation of double ICMs could occur during the early blastocyst stage; however, morphological observations alone are not sufficient to confirm whether the double ICMs observed in early blastocysts are actual ICMs. In this study, we performed OCT-4 staining in both hatching and hatched blastocysts. High frequencies of OCT-4-positive double ICMs were observed in ‘8’-shaped hatching blastocysts. The mechanism through which these double ICMs were formed in our study may be different from that in previous studies. Based on our work, we hypothesize that the ‘8’-shaped hatching is the most important factor affecting the formation of double ICMs. Herniation of the ICMs was easier to occur when the blastocysts were hatching through small openings in the zona pellucida, i.e. ‘8’-shaped hatching. A portion of the ICMs was split into two separate parts located in two blastocoels and sequentially formed double ICMs. According to our observations, in addition to the opening size, the position of the ICM relative to the zona pellucida opening is also critical for the formation of double ICMs. If the ICM is far away from the opening, ICM splitting is challenging, even if the opening size is small. This may explain why only 12.5% of OCT-4-positive double ICM was found in the ‘8’-shaped hatching blastocysts. Therefore, our study is the first report to illustrate that extended in vitro culture causes high frequency of ‘8’-shaped hatchings, leading to the risk of ICM splitting.

Over the past two decades, ART has been popularly used to achieve high rates of pregnancy in couples experiencing problems with fertility. However, the wide use of ART has been an important contributing factor to the increase in multiple birth rates [29,30]. Although the majority of multiple gestations produced by ART are dizygotic twins due to the replacement of more than one embryo, a growing body of evidence indicates that the wide use of ART, including intracytoplasmic sperm injection, assisted hatching, and extended embryo culture, could contribute to the increased occurrence of MZT [31–35]. The reason for this observation has been a matter of debate, and no definitive conclusion has been reached. Two case reports have presented two cases of ‘8’-shaped hatching human blastocysts with double ICM-like masses [21,22]. One of these cases resulted in a dizygotic trichorionic triamniotic triplet pregnancy when transferred together with another fully expanded blastocyst [21]. This type of ‘8’-shaped hatching from a human blastocyst during extended in vitro culture was consistent with our observations from a large number of mice ‘8’-shaped hatching blastocysts with small zona openings. However, zona alterations with atypical hatching alone are unlikely to account for all events of ICM splitting or MZT occurrence after blastocyst transfer. Study of monozygotic pregnancies following transfer of blastocysts without zona pellucida strongly suggests that other mechanisms, which are unrelated to alterations in the zona pellucida or the hatching process, may be involved [36].

## Conclusion

Our results indicate that extended in vitro culture causes a high frequency of ‘8’-shaped hatching and ‘8’-shaped hatching leads to the risk of ICM splitting in mouse blastocysts. More importantly, double ICMs within individual blastocysts were observed and verified in a large number of mouse blastocysts through immunofluorescence staining of OCT-4. Therefore, the present study provides at least one potential mechanism of the increased risk of ICM splitting in extended in vitro culture. However, further studies are needed to identify whether the ICM splitting caused by the ‘8’-shaped hatching during prolonged in vitro culture could lead to MZT birth after embryo transfer in mice. In addition, our data showed that ICMs disturbed or herniated by the small zona opening could impede the hatching process and increase the risk of abnormal ICMs. Therefore, we should pay more attention on the relationship between the hatching opening size of the zona pellucida and ICM splitting as well as pregnancy outcomes in human hatching blastocysts with or without micromanipulation.
